# Confirmed adult dengue deaths in Singapore: 5-year multi-center retrospective study

**DOI:** 10.1186/1471-2334-11-123

**Published:** 2011-05-12

**Authors:** Yee-Sin Leo, Tun L Thein, Dale A Fisher, Jenny G Low, Helen M Oh, Rajmohan L Narayanan, Victor C Gan, Vernon J Lee, David C Lye

**Affiliations:** 1 Department of Infectious Diseases, Tan Tock Seng Hospital, Singapore; 2 Communicable Disease Center, Tan Tock Seng Hospital, Singapore; 3 Department of Medicine, National University Hospital, Singapore; 4 Department of Internal Medicine, Singapore General Hospital, Singapore; 5 Department of Medicine, Changi General Hospital, Singapore; 6 Department of Medicine, Alexandra Hospital, Singapore; 7 Department of Clinical Epidemiology, Tan Tock Seng Hospital, Singapore; 8 Department of Epidemiology and Public Health, National University of Singapore, Singapore

## Abstract

**Background:**

Dengue re-emerges in Singapore despite decades of effective vector control; the infection predominantly afflicts adults. Severe dengue not fulfilling dengue hemorrhagic fever (DHF) criteria according to World Health Organization (WHO) 1997 guideline was increasingly reported. A new WHO 2009 guideline emphasized warning signs and a wider range of severe dengue manifestations. We aim to evaluate the utility of these two guidelines in confirmed adult dengue fatalities.

**Methods:**

We conducted a multi-center retrospective chart review of all confirmed adult dengue deaths in Singapore from 1 January 2004 to 31 December 2008.

**Results:**

Of 28 adult dengue deaths, median age was 59 years. Male gender comprised 67.9% and co-morbidities existed in 75%. From illness onset, patients presented for admission at a median of 4 days and death occurred at a median of 12 days. Intensive care admission was required in 71.4%. Probable dengue was diagnosed in 32.1% by WHO 1997 criteria and 78.6% by WHO 2009. The earliest warning sign was persistent vomiting at a median of 1.5 days. Hematocrit change ≥20% concurrent with platelet count <20 × 10^9/L was associated with the shortest interval to death at a median of 3 days. Only 35.7% of death cases fulfilled DHF criteria by WHO 1997 versus severe dengue in 100.0% by WHO 2009 criteria. Deaths were due to shock and organ failure. Acute renal impairment occurred in 71.4%, impaired consciousness 57.1% and severe hepatitis 53.6%.

**Conclusions:**

In our adult fatal dengue cohort, WHO 2009 criteria had higher sensitivity in diagnosing probable dengue and severe dengue compared with WHO 1997. As warning signs, persistent vomiting occurred early and hematocrit change ≥20% concurrent with platelet count <20 × 10^9/L preceded death most closely.

## Background

Dengue is the most important arthropod-borne viral disease in humans. The World Health Organization (WHO) has estimated that 1.8 billion people, or more than 70% of the global at-risk population, live in the WHO Southeast Asia and the Western Pacific regions which account for nearly 75% of current global disease burden from dengue [1]. Singapore, a developed island city-state in Southeast Asia has experienced resurgent dengue epidemics since the 1990s after previous decades of vector control effectively reduced the Aedes house index [2]. In addition, dengue infections in Singapore in recent years have shifted from primarily a childhood disease to that of adults [2]. With this resurgence, the classification of dengue cases for hospitalization and clinical management has gained importance.

The WHO 1997 guideline on management of dengue hemorrhagic fever (DHF) differentiates dengue manifestation into dengue fever (DF) and DHF, where plasma leakage underlies the pathophysiology of DHF [3]. This differentiation of dengue disease severity relied on data gathered from pediatric studies. Severe dengue especially in adults not fulfilling DHF criteria are being increasingly recognized [4]. Both Singapore and Puerto Rico reported confirmed adult dengue fatalities not fulfilling DHF and dengue shock syndrome (DSS) classification [5-7]. Persistent vomiting and abdominal pain were identified in a Cuban study as the most frequent warning signs [8], and tachycardia on admission was the only predictor of death in a Singapore case-control study [5]. In 2009, a new WHO guideline emphasized prominent gastro-intestinal symptoms in defining probable dengue and warning signs as predictors of severe dengue, and re-defined severe dengue beyond DHF and DSS [1].

This study describes a large cohort of adult dengue deaths in Singapore confirmed by dengue polymerase chain reaction (PCR) assay and non-structural protein 1 (NS1), and evaluates the utility of the two WHO guidelines 1997 and 2009 in confirmed adult dengue fatalities. This is important to determine the effectiveness of these guidelines in classifying dengue and severe dengue cases.

## Methods

We performed a multi-center retrospective observational study of adult dengue deaths at all five major adult public hospitals in Singapore from 1 January 2004 to 31 December 2008. Cases were identified through matching positive dengue diagnostic test results with hospital mortality records. Patients tested positive by dengue PCR or NS1 as previously described [9,10], and died within the same hospital admission, were included. Probable dengue diagnosed with rapid dengue serology was excluded due to lack of specificity [11].

Demographic, epidemiological, co-morbidity, serial clinical and laboratory, radiological, treatment and outcome data were obtained, and principal and secondary discharge diagnoses were recorded. The duration of acute illness was determined from the onset of fever, and from the onset of symptoms preceding hospitalization if fever was absent.

Institutional review boards of National Healthcare Group (Tan Tock Seng Hospital, National University Hospital and Alexandra Hospital) and Singhealth Group (Singapore General Hospital and Changi General Hospital), Singapore, approved the study, which was funded by the National Medical Research Council, Singapore. All records examined were anonymized.

According to the WHO 1997 guideline, DF clinically required the presence of fever and two or more of headache, retro-orbital pain, myalgia, arthralgia, rash, hemorrhagic manifestations and leukopenia; DHF the presence of all of fever, thrombocytopenia ≤100 × 10^9/L, any bleeding, and plasma leakage manifesting as either hematocrit change of ≥20%, clinical fluid accumulation (e.g. pleural effusion or ascites), or hypoproteinemia; and DSS the presence of one of rapid and weak pulse with narrow pulse pressure <20 mmHg, or hypotension for age in a patient with DHF.

Minor modifications to the WHO 2009 criteria were made in this study. Probable dengue was defined as fever and two of nausea or vomiting, rash, aches and pain, leukopenia, and any warning sign. The tourniquet sign was not performed in Singapore. Warning signs were determined as: abdominal pain or tenderness, persistent vomiting on at least two consecutive hospital days, clinical fluid accumulation (pleural effusion or ascites), mucosal bleed, lethargy or restlessness, any hepatomegaly (instead of greater than 2 cm), and hematocrit change of ≥20% with concurrent platelet nadir < 20 × 10^9/L or 50 × 10^9/L (instead of the qualitative rapid change in hematocrit with rapid drop in platelet count).

Severe dengue was present if any one of the following was recorded:

(1) Plasma leakage evidenced by hematocrit change of ≥20%, pleural effusion or ascites, or hypoproteinemia, leading to

(a) DSS (tachycardia, cold and clammy extremities, capillary refill time greater than three seconds, weak or undetectable pulse, narrow pulse pressure ≤20 mmHg, or systolic blood pressure <90 mmHg or unrecordable blood pressure), or

(b) Fluid accumulation with respiratory distress (respiratory rate ≥30/minute, oxygen saturation ≤92% on room air, or mechanical ventilation); or

(2) Severe bleeding manifesting as gastro-intestinal bleeding or menorrhagia, or requirement for transfusion of packed red blood cells or whole blood; or

(3) Severe organ involvement as follows:

(a) Serum alanine or aspartate transaminase ≥1000 units/L;

(b) Impaired consciousness;

(c) Acute renal impairment defined as serum creatinine > two times upper limit of normal [12]; or

(d) Myocarditis or encephalopathy/encephalitis.

For the statistical analysis, Fisher's exact test was applied to detect statistical difference between dichotomous variables, while the Kruskal-Wallis test was used to test for significant trend for continuous data. The analysis was performed in SPSS version 16 (SPSS Inc., Chicago, IL, USA), with the level of significance set at a two-tailed *p *value of <0.05.

## Results

### Clinical and epidemiological features of the cohort

There were 27 adult dengue deaths confirmed by dengue PCR and one by NS1 from 1 January 2004 to 31 December 2008 in the five Singapore adult public hospitals - six in 2004, nine in 2005, three in 2006, eight in 2007 and two in 2008. The lowest estimated incidence of adult dengue death was 6.8 per 10,000 dengue cases, based on 41,234 notified dengue cases over this period [13] (potential dengue deaths outside of the five hospitals and those diagnosed with dengue serology were not included). Males comprised 19 (67.9%) of the deaths and the median age was 59 years (range, 21-86 years). Co-morbidities existed in 21 (75.0%) deaths; with hypertension in 12, cardiovascular diseases in 10, diabetes mellitus in 9, nephropathy in 8, chronic lung diseases in 4, and 3 each with cancer, corticosteroid use and hyperlipidemia, and 1 with liver disease.

Admission diagnoses recorded in the medical notes in emergency department were dengue and dengue-related in eight cases; namely dengue (6 cases), DSS (1) and viral fever (1). The median duration from illness onset to hospital admission was 4 days (range, 1-10 days), from admission to the taking of the positive dengue test sample was 2.5 days (range, 1-11 days). Twelve patients survived more than seven days after positive dengue test, of which five of them survived more than 2 weeks. The median duration from illness onset to death was 12 days (range, 2-38 days) and from the positive dengue test to death was 6 days (range, 1-32 days). The median length of hospital stay was 8.5 days (range, 2-34 days); fifteen patients were hospitalized for more than seven days and seven patients more than 14 days before death. Two of those seven cases developed bacteremia. Dengue was stated as primary cause of death or primary discharge diagnosis in 16 subjects - 6 were diagnosed as DSS, 6 DHF and 4 DF. Autopsy was not performed in any patient.

Twenty (71.4%) patients required admission to intensive care units (ICU), three of whom directly from emergency department. The median duration from hospital to ICU admission was 3 days (range, 1-9 days). The median duration of ICU stay was 3 days (range, 1-32 days). For the entire cohort, mechanical ventilation was needed in 4 cases, inotropic support 14, blood transfusion 8, and antibiotic 23.

Bacteremia was documented for four (14.3%) patients at a median duration of 6.5 days (range, 2-22 days) from hospital admission and 1.5 days (range, 1-21 days) from dengue diagnosis. These included *Pseudomonas aeruginosa *(1), *methicillin-resistant Staphylococcus aureus *(2), and alpha-hemolytic streptococcus (1).

### Clinical features at presentation and within seven days of dengue diagnosis

Reported fever was present in 21 (75.0%) at presentation, increasing to 25 (89.3%) around the time of dengue diagnosis. Recorded temperature of ≥37.5°C was present at presentation in 19 (67.9%) and ≥38.0°C in 13 (46.4%) cases, and within seven days of dengue diagnosis in 25 (89.3%) and 21 (75%) cases respectively. Given the older age of our cohort, the lower temperature cut-off of ≥37.5°C was more sensitive. Taken together, reported fever or recorded temperature of ≥37.5°C was present in 24 (85.7%) at presentation, increasing to 27 (96.4%) within seven days of dengue diagnosis.

Table 1 showed the diagnostic criteria for probable dengue according to the WHO 1997 and 2009 guidelines. At presentation, the WHO 1997 criteria for probable dengue were fulfilled in only 4 (14.3%) cases, and the WHO 2009 criteria in 17 (60.7%) cases. Within seven days of dengue diagnosis, the WHO 1997 was present in 9 cases (32.1%) versus WHO 2009 in 22 (78.6%).

**Table 1:** Relevant clinical and laboratory data at hospital presentation and within seven days of dengue diagnosis

	No.(%) of patients at presentation	No.(%) of patients within seven days of positive dengue	p*
WHO 1997 criteria for probable dengue			
Fever/temperature ≥37.5°C plus two or more of the following:	4 (14.3)	9 (32.1)	0.205
Fever:			
Fever symptom	21 (75.0)	25 (89.3)	0.296
Temperature ≥37.5°C	19 (67.9)	25 (89.3)	0.101
Symptom/temperature ≥37.5°C	24 (85.7)	27 (96.4)	0.352
Headache	3 (10.7)	4 (14.3)	1.000
Retro-orbital pain	0 (0.0)	0 (0.0)	na
Myalgia	7 (25.0)	9 (32.1)	0.768
Arthralgia	0 (0.0)	2 (7.1)	0.491
Rash	1 (3.6)	3 (10.7)	0.611
Hemorrhagic manifestation	5 (17.9)	6 (21.4)	1.000
Leukopenia	6 (21.4)	11 (39.3)	0.245
			
WHO 2009 criteria for probable dengue			
Fever symptom/temperature ≥37.5°C and two of the following:	17 (60.7)	22 (78.6)	0.245
Fever symptom/temperature ≥37.5°C	24 (85.7)	27 (96.4)	0.352
Nausea or vomiting	10 (35.7)	10 (35.7)	na
Rash	1 (3.6)	3 (10.7)	0.611
Aches and pain	18 (64.3)	19 (67.9)	1.000
Leukopenia	6 (21.4)	11 (39.3)	0.611
Any warning sign	19 (67.9)	27 (96.4)	0.012
			
WHO 2009 criteria for warning signs			
Abdominal pain or tenderness	9 (32.1)	10 (35.7)	1.000
Persistent vomiting ≥2 days	0 (0.0)	2 (7.1)	0.491
Clinical fluid accumulation	2 (7.1)	13 (46.4)	0.002
Mucosal bleeding	0 (0.0)	4 (14.3)	0.112
Lethargy	11 (39.3)	16 (57.1)	0.285
Hepatomegaly	2 (7.1)	2 (7.1)	na
Hematocrit change ≥20%	9 (32.1)	25 (89.3)	<0.001
Hematocrit change ≥20% with platelet <50 × 10^9/L	1 (3.6)	20 (71.4)	<0.001
Hematocrit change ≥20% with platelet <20 × 10^9/L	1 (3.6)	14 (50.0)	<0.001
Any warning sign	19 (67.9)	27 (96.4)	0.012

na = not applicable, WHO = World Health Organization

*Fisher's exact test

The commonest warning sign at presentation according to the WHO 2009 criteria was lethargy in 11 (39.3%), and hematocrit change of ≥20% with concurrent platelet count of <50 × 10^9/L was the commonest (20 [71.4%]) within seven days of dengue diagnosis (Table 1). Significant increase from hospital presentation to within seven days of dengue diagnosis was noted for fluid accumulation (from 3 [7.1%] to 13 [46.4%], *p *= 0.002), hematocrit change of ≥20% with concurrent platelet count of <50 × 10^9/L (from 1 [3.6%] to 20 [71.4%], *p *< 0.001) and concurrent platelet count of <20 × 10^9/L (from 1 [3.6% to 14 [50.0%], *p *< 0.001), and any warning sign (from 19 [67.9%] to 27 [96.4%], *p *= 0.012).

**Figure 1:**
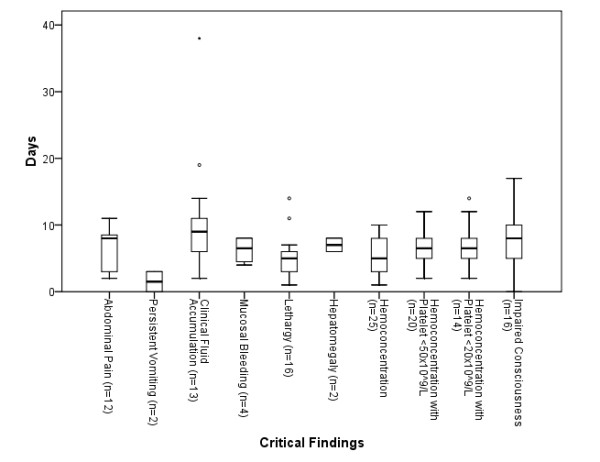
**Interval from illness onset to warning signs**. Kruskal-Wallis test *p *< 0.05 (Circles represents ≥1.5 times Inter Quartile Range [IQR] and dot represents ≥3 times IQR from median)

**Figure 2:**
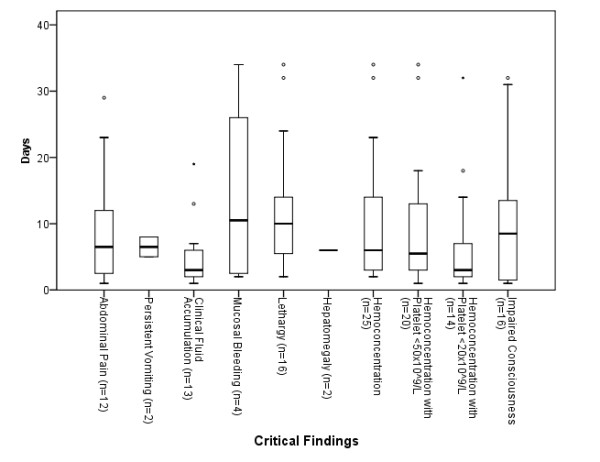
**Interval from onset of warning signs to death**. Kruskal-Wallis test *p *> 0.05 (Circles represents ≥1.5 times Inter Quartile Range [IQR] and dot represents ≥3 times IQR from median)

Figures 1 and 2 show the interval from illness onset to developing warning signs, and from developing warning signs to death respectively. The interval from illness onset to developing warning signs was shortest for persistent vomiting (median 1.5 days), followed by lethargy and hematocrit change of ≥20% (median 5 days). The interval from developing warning signs to death was shortest for hematocrit change of ≥20% with concurrent platelet count of <20 × 10^9/L (median 3 days). The difference in intervals from illness onset to developing warning signs was statistically significant, but not for the intervals from developing warning signs to death.

### Comparison of WHO 1997 and 2009 criteria for defining severity of dengue

In the WHO 1997 guideline, severe dengue was mainly defined as DHF and DSS, with plasma leakage as the underlying pathogenesis. As shown in Table 2, DHF criteria were only fulfilled in 10 (35.7%); fever was present in 27 (96.4%), thrombocytopenia 26 (92.9%), plasma leakage 28 (100%) and bleeding 10 (35.7%). If bleeding was excluded, the WHO 1997 criteria would be fulfilled in all cases. Dengue shock syndrome defined in the WHO 1997 criteria was present in 8 (28.6%). In comparison, severe dengue defined by the WHO 2009 criteria was fulfilled in 28 (100.0%). Among the categories of severe dengue defined by the WHO 2009 guideline (Table 3), renal impairment was the commonest (20 [71.4%]), followed by impaired consciousness (16 [57.1%]) and severe elevation of transaminases ≥1000 units/L (15 [53.6%]). Hypoproteinemia was present in 25 (89.3%) but only two cases did not have hematocrit change ≥20% or fluid accumulation. The WHO 2009 guideline classified plasma leakage with tachycardia (pulse was >100/minute in 26 [92.9%] and >120/minute in 21 [75.0%]) as DSS but this accounted for only two cases (Table 3).

**Table 2:** WHO 1997 criteria for dengue hemorrhagic fever (DHF) and dengue shock syndrome (DSS)

Criteria	No.(%) of patients
DHF (fulfilling all 4 criteria):	10 (35.7)
DHF (fulfilling 3 criteria excluding bleeding):	28 (100.0)
Fever	27 (96.4)
Platelet ≤100 × 10^9/L	26 (92.9)
Any bleeding	10 (35.7)
Plasma leakage:	28 (100.0)
Hematocrit change ≥20%	25 (89.3)
Pleural effusion or ascites	13 (46.4)
Hypoproteinemia	25 (89.3)
DSS:	8 (28.6)
DHF, rapid pulse and narrow pulse pressure	5 (17.9)
Rapid pulse >100/minute	26 (92.9)
Narrow pulse pressure <20 mmHg	10 (35.7)
DHF and hypotension	8 (28.6)
Hypotension <90 mmHg	21 (75.0)

WHO = World Health Organization

**Table 3:** WHO 2009 criteria for severe dengue

Criteria	No.(%) of patients
Severe dengue	28 (100.0)
Severe plasma leakage (hematocrit change ≥20%, fluid accumulation, hypoproteinemia)	28 (100.0)
Severe plasma leakage (hematocrit change ≥20%, fluid accumulation)	26 (92.9)
Shock:	
Shock (tachycardia or narrow pulse pressure or hypotension)	28 (100.0)
Shock (narrow pulse pressure or hypotension)	26 (92.9)
Tachycardia >100/minute	26 (92.9)
Narrow pulse pressure <20 mmHg	10 (35.7)
Hypotension <90 mmHg	21 (75.0)
Fluid accumulation with respiratory distress	9 (32.1)
Significant bleeding:	10 (35.7)
Gastrointestinal tract	3 (10.7)
Menorrhagia	0 (0.0)
Transfusion of packed red cells or whole blood	8 (28.6)
Impaired consciousness:	16 (57.1)
Coma	1 (3.6)
Drowsiness	13 (46.4)
Confusion	4 (14.3)
Severe gastrointestinal involvement:	3 (10.7)
Persistent vomiting ≥2 days	2 (7.1)
Jaundice	3 (10.7)
Severe organ involvement:	24 (85.7)
ALT or AST ≥1000 units/L	15 (53.6)
Impaired consciousness	16 (57.1)
Serum creatinine >2x upper limit normal	20 (71.4)
Myocarditis	0 (0.0)
Encephalitis	0 (0.0)

ALT = alanine transaminase, AST = aspartate transaminase, WHO = World Health Organization

## Discussions and Conclusions

Singapore experienced large dengue epidemics in the past decade affecting predominantly adults [13]. This shift from childhood to adult illness was attributed to lower herd immunity and transmission outside home [2]. Epidemiological data showed adults were at lower risk of DHF than children [2,14]. Identifying dengue cases, and severe dengue cases among them, are important to allow for targeted clinical management and close monitoring. Difficulties in applying the WHO 1997 DHF criteria reported by many countries led to a new WHO guideline in 2009 focussing on recognizing warning signs and re-defining severe dengue [1,4,15,16]. We found that the diagnostic criteria for probable dengue from the WHO 2009 guideline identified 78.6% of our adult confirmed dengue deaths compared with 32.1% for the WHO 1997 criteria. In addition, throughout the hospital admissions, the WHO 1997 DHF criteria could only be fulfilled in 35.7% of cases, mainly due to the lack of clinical bleeding, while the WHO 2009 criteria classified all cases as severe dengue.

As for the importance of individual warning signs in dengue deaths, a retrospective study in Puerto Rico identified abdominal pain, persistent vomiting, abrupt temperature change and abnormal mental status as four major warning signs [7]. Similarly, in Cuba all twelve fatalities had headache, persistent vomiting, malaise, bleeding and shock [8]. In another Singapore study, abdominal pain, and nausea and vomiting occurred in half of adult dengue deaths [5]. In our study, the presence of any warning sign was the commonest diagnostic criterion at hospital presentation (67.9%) apart from fever, and within seven days of dengue diagnosis (27 [96.4%]). For individual warning signs, lethargy was the commonest at hospital presentation (39.3%) but within seven days of dengue diagnosis, hemoconcentration with concurrent thrombocytopenia <50 × 10^9/L became the commonest (71.4%).

Our study also showed that impaired consciousness increased from 7 (25.0%) at hospital presentation to 16 (57.1%) within seven days of dengue diagnosis. Rigau-Perez et al reported a high proportion of impaired consciousness at presentation and during disease progression [7]. Other studies showed that dengue encephalopathy was diagnosed in 0.5% of children with DHF, and that impaired consciousness was associated with high mortality and multi-organ involvement [17,18]. The importance in recognizing this sign in potentially severe dengue should be emphasized.

Hemoconcentration was documented in 89.3% of our patients while Cuba reported a similarly high 91.6% [8]. The WHO 2009 criteria lack clarity in quantifying the degree and rate of change in hematocrit and platelet count [1]. The combination of hematocrit change ≥20% and concurrent thrombocytopenia <50 × 10^9/L and <20 × 10^9/L were present in 71.4% and 50.0% of our cases respectively. Our findings provide clinicians with additional quantitative criteria as part of the new WHO warning signs.

Our study also detailed the interval from illness onset to developing warning signs and progression to death. Persistent vomiting ≥ 2 days was the earliest warning sign in our study followed by lethargy and hemoconcentration. Notably, rapid progression to death in our cohort occurred once hemoconcentration with thrombocytopenia developed. In Cuba, vomiting was also one of the commonest warning signs, and deterioration occurred at a mean of 3.75 days from illness onset with total illness duration of 7 days [8]. Hence, clinicians should identify dengue patients with persistent vomiting ≥2 days for close monitoring for hematocrit change ≥20% concurrent with thrombocytopenia <20 × 10^9/L.

There were three earlier reports from Singapore on adult dengue deaths. The age of the deceased in our study and two recent studies [5,6] was older than a much earlier report by Chan et al [19] with possible shift of severe disease to older population. In our study, 75% had co-morbidities similar to other studies [7,8]. Asthma, diabetes mellitus and sickle cell disease have been reported risk factors for DHF [20] and await validation in well-designed case control studies. In Cuba white race was prevalent among fatal cases [8], but in multi-racial Singapore, we found no evidence of ethnic preponderance.

Our study included many elderly dengue deaths with a median age of 59 years. Two studies on dengue in older adults suggested fewer constitutional symptoms in these patients [21,22]. Dengue shock syndrome was an independent risk factor for mortality among the elderly in Taiwan [21]. Multi-organ failure with renal impairment was common in older patients [23]. A study from Taiwan reported that concurrent bacteremia occurred in 7.3% of all study subjects compared with 17.4% in the elderly [21]. Bacteremia was detected in 14.3% in our study, possibly due to older patients and prolonged hospitalization (53.6% of our patients were hospitalized for more than a week and 25% more than two weeks). Recognizing and treating nosocomial infections in complicated dengue infection should therefore be emphasized.

There are some limitations of our retrospective study as it depended on the thoroughness of clinicians' documentation. Documentation on bleeding including petechiae was frequently missing, undermining the utility of WHO 1997 DHF criteria [24]. We did not perform the tourniquet test, which is part of WHO criteria for hemorrhagic tendency. A Vietnamese study found that tourniquet provided additional diagnostic information only in 5% of cases [25]. Information on dengue sero-type and primary versus secondary dengue infection were lacking. However, a prospective study may not be feasible given infrequent adult dengue death, and our study provides a reflection of the possible implications of use of the two WHO criteria, and additional warning signs to be emphasized. Our findings in a highly selected sample of adult dengue fatalities need to be validated in less severe form of dengue diseases in both adults and children. The predominance of older adults in this study raises the need to include older individuals in future vaccine studies.

In conclusion, our findings showed that the WHO 2009 guideline was effective in diagnosing probable and severe dengue in adult dengue deaths. We have also quantified the change in hematocrit and platelet count as part of WHO 2009 warning signs, and have suggested that impaired consciousness may be an important warning sign.

Word count = 2856

## Competing interests

YS Leo is medical advisor to Sanofi-Pasteur on a dengue vaccine trial. HM Oh is an investigator of Sanofi-Pasteur dengue vaccine trial. VJ Lee received unrelated research funding from GSK.

## Authors' contributions

YSL, JGL, HMO and DCL contributed to the study conception and design. YSL, DAF, JGL, HMO and RLN contributed to the acquisition of data. YSL, TLT, VCG and DCL analysed and interpreted the data. YSL wrote the first draft of the manuscript. TLT, DCL and VJL revised the manuscript. All authors read and approved the final manuscript.

## Pre-publication history

The pre-publication history for this paper can be accessed here:

http://www.biomedcentral.com/1471-2334/11/123/prepub
